# Parkinson’s Disease: Biomarkers for Diagnosis and Disease Progression

**DOI:** 10.3390/ijms252212379

**Published:** 2024-11-18

**Authors:** Rakesh Arya, A. K. M. Ariful Haque, Hemlata Shakya, Md. Masum Billah, Anzana Parvin, Md-Mafizur Rahman, Khan Mohammad Sakib, Hossain Md. Faruquee, Vijay Kumar, Jong-Joo Kim

**Affiliations:** 1 Department of Biotechnology, Yeungnam University, Gyeongsan 38541, Republic of Korea; rakesharya101@yu.ac.kr; 2 Department of Biotechnology and Genetic Engineering, Faculty of Biological Sciences, Islamic University, Kushtia 7003, Bangladesh; ariful.btge20.iu@gmail.com (A.K.M.A.H.); masumbillah111998@gmail.com (M.M.B.); anzana@btge.iu.ac.bd (A.P.); mmrahman@btge.iu.ac.bd (M.-M.R.); faruquee@btge.iu.ac.bd (H.M.F.); 3 Department of Biomedical Engineering, Shri G. S. Institute of Technology and Science, Indore 452003, India; hemlata.shakya19@gmail.com; 4 Department of Biology, Adamjee Cantonment College, Dhaka Cantonment, Dhaka 1206, Bangladesh; khan11sakib@gmail.com; 5 Department of Orthopaedic Surgery, The Johns Hopkins University School of Medicine, Baltimore, MD 21205, USA

**Keywords:** Parkinson’s disease, biomarker, biochemical, neuroimaging, genetic, α-synuclein

## Abstract

Parkinson’s disease (PD) is a progressive neurological disease that causes both motor and nonmotor symptoms. While our understanding of putative mechanisms has advanced significantly, it remains challenging to verify biomarkers with sufficient evidence for regular clinical use. Clinical symptoms are the primary basis for diagnosing the disease, which can be mild in the early stages and overlap with other neurological disorders. As a result, clinical testing and medical records are mostly relied upon for diagnosis, posing substantial challenges during both the initial diagnosis and the continuous disease monitoring. Recent biochemical, neuroimaging, and genetic biomarkers have helped us understand the pathophysiology of Parkinson’s disease. This comprehensive study focuses on these biomarkers, which were chosen based on their relevance, methodological excellence, and contribution to the field. Biochemical biomarkers, including α-synuclein and glial fibrillary acidic protein (GFAP), can predict disease severity and progression. The dopaminergic system is widely used as a neuroimaging biomarker to diagnose PD. Numerous genes and genome wide association study (GWAS) sites have been related to the development of PD. Recent research on the SNCA gene and leucine-rich repeat protein kinase 2 (LRRK2) has shown promising results. By evaluating current studies, this review intends to uncover gaps in biomarker validation and use, while also highlighting promising improvements. It emphasizes the need for dependable and reproducible indicators in improving PD diagnosis and prognosis. These biomarkers may open up new avenues for early diagnosis, disease progression tracking, and the development of personalized treatment programs.

## 1. Introduction

The World Health Organization issued a technical brief titled “Parkinson’s disease: a public health approach” in June 2022, emphasizing that the worldwide effect of Parkinson’s disease (PD) is “increasing faster than for any other neurological disorder”. According to the paper, the prevalence of PD has more than doubled in the last 25 years, with over 5.8 million disability-adjusted life years in 2019—an 81% rise since 2000 [1].

PD is a neurodegenerative disorder that affects the central nervous system and it affects dopamine-producing neurons in the substantia nigra region of the brain [2]. The accumulating intraneuronal aggregates of the protein α-synuclein, also known as Lewy bodies, and the gradual degradation of dopaminergic neurons in the substantia nigra are the main pathogenic alterations observed in PD patients [3]. It is a type of disorder that occurs when the brain contains an insufficient amount or quantity of dopamine. Since dopamine is linked to motor activity, dopamine depletion is the major cause of PD. Tremors, slowness and paucity of movement, stiffness or the slowing of movements, and difficulty in walking, talking, or doing other simple tasks are common symptoms that develop slowly over time [4]. The primary method for diagnosing PD is a clinical assessment. The primary symptom for diagnosis is bradykinesia, which is accompanied by at least one additional symptom such as resting tremor, rigidity, or postural instability. Clinicians must gather a patient’s medical history and conduct a thorough neurological examination to exclude other medical conditions that may resemble PD [5,6]. The Movement Disorder Society (MDS) has published criteria for classifying PD as clinically established or likely based on the existence of supporting and exclusion criteria. Imaging techniques such as dopamine transporter scans and MRIs can help with the diagnosis, although they are not precise [6]. Nonmotor symptoms can occur along with other parkinsonism, including atypical parkinsonisms. Dysautonomia, a nonmotor symptom, is particularly significant in both PD and atypical parkinsonisms. Recent research emphasizes the significance of treating both motor and autonomic dysfunction to accurately differentiate between these diseases [7]. Furthermore, nonmotor symptoms such as cognitive impairment, disturbances in sleep, and mood disorders are widely established symptoms of PD [8]. PD can be treated symptomatically, typically with medication that targets both motor and nonmotor symptoms. The therapeutic landscape has developed dramatically, with a focus on symptom management to improve a patient’s quality of life [9,10].

PD is incurable; however, there are numerous treatment options available, and it can be treated symptomatologically. Although some cases are hereditary and can be traced back to specific genetic mutations, the majority of cases are sporadic and are likely to be the consequence of a mix of heredity and exposure to one or more environmental factors [11,12]. The rate of PD misdiagnosis by movement disorder specialists is high, due, in part, to the lack of sensitive and precise biomarkers established for physicians to differentiate PD from other movement disorders with overlapping clinical symptoms [13]. Over the last five years, clinical diagnostic criteria aiming to improve the diagnostic accuracy of PD have been validated. However, diagnosing PD is challenging because its clinical symptoms frequently overlap with those of other neurodegenerative disorders, and current tests or biomarkers do not allow for a conclusive diagnosis in the early stages [14]. Consequently, even when the disease is fully apparent clinically, the accuracy of clinical diagnosis remains insufficient. Aside from diagnostic utility, biomarkers for PD are required to monitor disease progression and the efficacy of therapies, both of which are currently measured by assessing the severity of the motor symptoms.

In this review, we summarize what is currently known about PD biomarker research from biochemical, genetic, and neuroimaging perspectives, including the most recent advancements, their diagnostic accuracy, and potential uses. This review aims to expand our knowledge and understanding of biomarkers and Parkinson’s disease broadly and discuss our suggestions for the future.

## 2. Methodology

The purpose of this review is to investigate the numerous biomarkers linked with PD, bringing together information gathered from biochemical, neuroimaging, and genetic studies. The research question motivating this study is: “What are the key biomarkers of Parkinson’s disease that show promising results with respect to diagnosis, as identified through biochemical, neuroimaging, and genetic research?”. To address this question, a complete synthesis of the available information is required, hopefully guiding future studies.

### 2.1. Inclusion and Exclusion Criteria

The literature search was undertaken using numerous databases, including PubMed, Scopus, and Web of Science, with an emphasis on research published within the previous decade.

Inclusion Criteria

- Peer-reviewed studies examining biochemical, neuroimaging, or genetic biomarkers associated with Parkinson’s disease.
- Articles providing important and new insights into the mechanisms of these biomarkers.

Exclusion Criteria

- Non-peer-reviewed papers, research on unrelated neurological disorders, and articles with outdated and insufficient information were all excluded.
- The purpose of these selections is to provide a high-quality synthesis that captures the key research while remaining relevant to the review.

### 2.2. Details of Analysis and Interpretation

The analysis involved a thematic synthesis of the findings from the selected studies. Each category of biomarkers is discussed separately, summarizing recent key findings while identifying patterns and discrepancies across studies.

- Biochemical analysis: this section explores specific proteins identified as potential biomarkers, discussing their roles in pathophysiology and diagnostic utility.
- Neuroimaging insights: here, various imaging modalities are evaluated with respect to their effectiveness in detecting early changes associated with Parkinson’s disease.
- Genetic factors: this part focuses on the genetic markers associated with an increased risk or familial forms of Parkinson’s disease.

The interpretation highlights how these biomarkers contribute to the understanding of disease mechanisms, improving diagnostic accuracy and, potentially, guiding therapeutic strategies.

## 3. Biomarkers for Parkinson’s Disease

**Figure 1:**
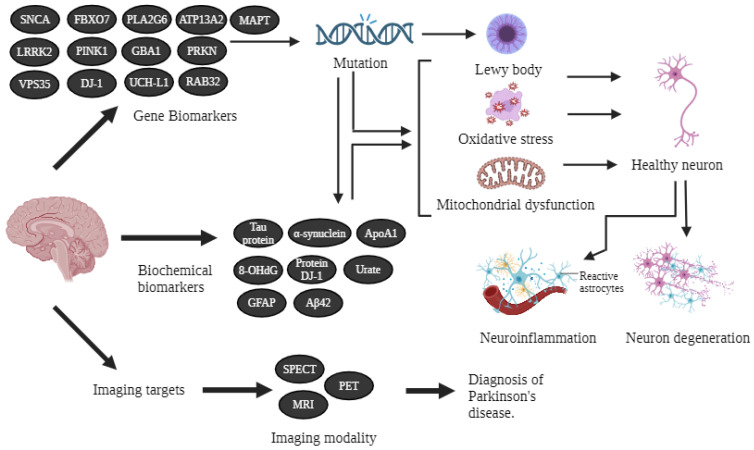
Schematic representation of PD biomarkers. The three primary categories of PD biomarkers are genetic, biochemical, and neuroimaging biomarkers, each leading to insights into disease mechanisms and progression. Created with www.BioRender.com.

A biomarker is a characteristic that can be assessed as an indicator of normal biological pathogenic processes or of responses to different exposures or interventions, including therapeutic interventions [15]. The early phases of the disease, when treatment is most effective, require the use of biomarkers. Indicators such as imaging, cerebrospinal fluid, oxidative stress, inflammation, and neuroprotection can help with the early identification of the disease [16]. The quest for biomarkers of PD is an ongoing endeavor [17]. Clinical, imaging, biochemical, and genetic biomarkers are the four types of biomarkers [18]. Individual biomarker specificity and sensitivity are not perfect, and the combination of multimodal biomarkers is likely to improve PD diagnostic accuracy. Efforts to analyze and validate putative biomarkers are uncommon, and more research is needed to identify accurate biomarkers for early disease detection and management [17] (Figure 1).

### 3.1. Biochemical Biomarkers

Orexin is known to be involved in narcolepsy, but increasing evidence suggests that it may also be involved in other neurodegenerative conditions, such as Parkinson’s, Alzheimer’s, epilepsy, amyotrophic lateral sclerosis, and Huntington’s disease [19]. A recent study has examined how orexin-A promotes dopaminergic neuron survival and function by working on orexin receptors. Orexin-A has been demonstrated to slow the death of dopaminergic neurons while also improving cognitive and motor functions, indicating that it has therapeutic promise in the context of PD [20]. Another study states that changes in orexin levels may have a role in the pathophysiology of nonmotor symptoms such as sleep disturbances and cognitive impairment. Notably, elevated plasma levels of orexin-A have been observed in individuals with early-stage PD, suggesting that orexin-A may function as a compensation mechanism against inflammatory and oxidative stress [21]. Orexin receptor antagonists may help control sleep disturbances, which are common in PD patients; however, there is concern around the fact that they may worsen both motor and nonmotor symptoms by blocking orexin signaling pathways, which are critical for maintaining dopaminergic neuron function [22]. The intermediate filament protein glial fibrillary acidic protein (GFAP) is mostly expressed in central nervous system astrocytes. When there is damage to the nervous system, astrocytes quickly release GFAP into the circulation [10]. A few studies looking at serum GFAP in PD patients have found higher levels of GFAP in the serum than in the controls [23,24]. Astrocytes produce the inflammatory marker known as glial fibrillary acidic protein, or GFAP. Research has demonstrated that plasma GFAP can indicate when dementia will start to develop in PD patients with mild cognitive impairment (PD-MCI) [25]. The type-III intermediate filament, known as glial fibrillary acidic protein (GFAP) and mostly expressed by astrocytes, can be used to identify reactive astrogliosis. The expression of PD and Alzheimer’s disease (AD) is significantly heightened, both being notable neurodegenerative disorders [26,27].

The oxidation of DNA nucleosides generates 8-hydroxy-2-deoxyguanosine (8-OHdG), which serves as a valuable indicator of oxidative stress. There is evidence that the cerebrospinal fluid (CSF) of patients suffering from a range of neurodegenerative diseases has higher than normal levels of oxidative stress. A neurochemically supported diagnosis of PD may benefit from the measurement of 8-OHdG levels [28]. The possibility of polymorphism and PD altering biochemical measures has been investigated in a recent study. Parameters of oxidative stress, including 8-OHdG, ROS, and NADPH, have been shown to be strongly correlated with the risk of PD [29]. The α-synuclein (αSyn) seed amplification assay (SAA) identifies αSyn aggregates as a sign of Lewy body pathology in patient-derived biomaterials, aiding the diagnosis of dementia syndromes and PD [30]. Misfolded αSyn aggregates, or seeds, have an inherent self-replicative characteristic that allows (αSyn-SAAs) for their proliferation in vitro. In these tests, aggregate fragmentation into smaller self-propagating seeds and elongation at the expense of recombinant αSyn (rec-αSyn) are the two cyclical processes that increase the amount of αSyn seeds circulating in bodily fluids. Fluorescent dyes unique to amyloids, like thioflavin T, can detect the seeds after they have been amplified [31]. Larger analyses of the αSyn-SAA for the biochemical diagnosis of PD show that the assay classifies patients suffering from PD with high sensitivity and specificity, provides information about molecular heterogeneity, and detects prodromal individuals before diagnosis [32]. According to the results of a recent study, developing disease-modifying therapies to stop the spread of pathological proteins in PD and Alzheimer’s disease (AD) may benefit from focusing on particular tau and aSyn variants with elevated post translational modification (PTM), such as pS129 aSyn and C-terminus epitopes [33]. The amount of alpha-synuclein in PD patients’ saliva can vary, something which could be valuable for the early detection of this neurodegenerative disease and for its distinction from other synucleinopathies [34].

Reduced Apo A1 levels have been linked to an increased risk of PD, as well as to a more severe form of the disease and to the manifestation of PD at an earlier age [35]. A recent study using a machine learning technique has demonstrated the substantial correlation among TG, ApoA1, SNCA (rs6826785), and PD-MCI [36]. A related study has shown that, whilst PD-MCI patients have greater levels of TC, TG, and Apo A1, PD patients have considerably lower levels of lipid biomarkers. For PD-MCI patients, TC, TG, and Apo A1 might be helpful biomarkers [37].

Aβ42 plays a role in the build-up of amyloid plaques, a hallmark of AD. However, Aβ42 has a more nuanced function with respect to PD [38]. In contrast, certain research studies have indicated that PD patients experiencing cognitive decline have a higher Aβ42 burden [39]. PD with a significant probability of worsening may be identified by tau, Aβ42, and plasma EV α-synuclein combined. The results of a new study can clarify how these pathognomonic proteins interact, and they may be used in therapeutic strategies to modify disease progression or as indicators of therapy response [40].

**Figure 2:**
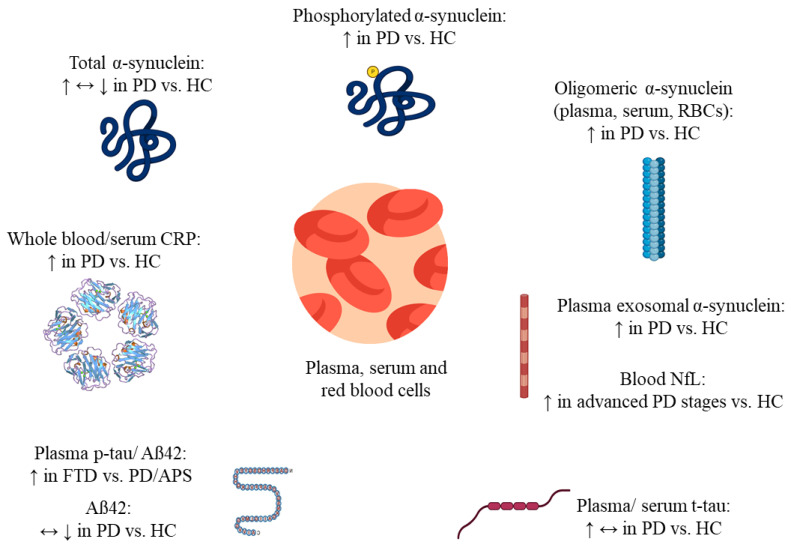
Possible blood-based biomarker of Parkinson’s disease. This figure provides evidence supporting the potential of the blood-based biomarker for Parkinson’s disease, shows significant differences in levels between PD patients and controls, good diagnostic accuracy, correlation with disease severity, dynamic changes over time, and reproducibility in an independent cohort. Total α-synuclein levels show inconsistent results, with some studies reported increase (↑), others decrease (↓), and some no significant difference (↔) between PD patients and HC. This variability may stem from differences in sample sources or detection methods. Phosphorylated α-synuclein is consistently elevated (↑) in PD patients as compared to HC. Levels of oligomeric forms of α-synuclein are increased (↑) in PD patients' plasma, serum, and red blood cells, which is more closely associated with neurotoxicity and PD pathology. Exosomal α-synuclein is also elevated (↑) in PD patients compared to HC, supporting its role as a biomarker for PD. Blood NfL levels are significantly higher (↑) in advanced stages of PD, reflecting neurodegeneration and disease progression. Tau protein levels, both in plasma and serum, are generally increased (↑) in PD, but other studies report no significant change (↔). Aβ42 levels in PD are unchanged (↔) or reduced (↓) compared to HC, reflecting study variability and potential differences in disease mechanisms. Levels of phosphorylated tau (p-tau) and Aβ42 are increased (↑) in FTD patients compared to PD or APS. CRP protein levels, both in whole blood and serum, are generally elevated (↑) in PD patients as compared to HC. PD; Parkinson’s disease, HC; Healthy control, FTD; Frontotemporal dementia, APS; Atypical parkinsonian syndromes, CRP; C-Reactive protein, Aβ42; amyloid-beta 42, NfL; Neurofilament light chain, ↑; upregulated, ↓; downregulated, ↔; unchanged.

**Figure 3:**
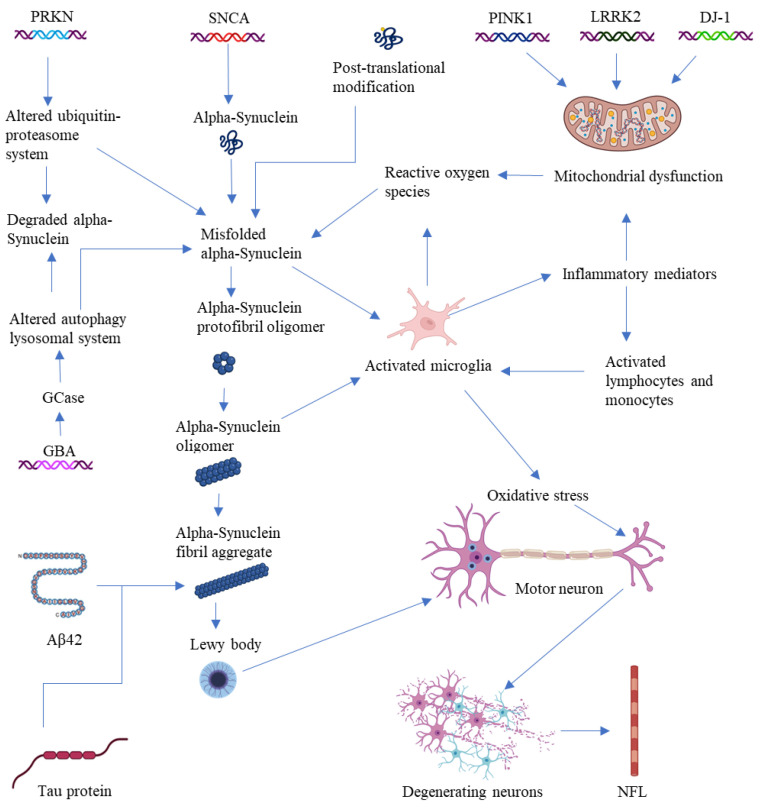
Cerebrospinal fluid (CSF) biomarkers of PD. This figure illustrates the complex pathways involved in PD pathology and highlights key cerebrospinal fluid (CSF) biomarkers associated with the disease. The molecular mechanisms underlying PD facilitate early diagnosis and the development of targeted therapies.

Mutations in the DJ-1 protein generate autosomal recessive variations, and oxidized DJ-1 has been found in the brains of patients with idiopathic PD [41]. Early-onset PD with autosomal recessive inheritance is caused by mutations in PARK7 that result in nonfunctional, or in the total loss of, DJ-1 protein. In general, PARK7/DJ-1 dysfunction significantly impact the immunological responses and neuroinflammatory processes of microglia [42]. Low serum UA levels may correlate with the severity of sleep disorders in PD patients and act as a biomarker for poor sleep quality in the context of PD [43]. Low levels of serum uric acid are associated with nonmotor symptoms of PD [44]. In vitro, tau-modified α-synuclein fibrils show higher seeding activity than pure α-synuclein fibrils, resulting in mitochondrial malfunction, synaptic impairment, and neurotoxicity [45]. Tau-PET imaging enables the visualization and measurement of tau load in the brain, offering insights into the distribution and impact of tau pathology with respect to PD patients [46] (Figure 2 and Figure 3, Table 1).

### 3.2. Neuroimaging Biomarkers

Neuroimaging biomarkers are essential for PD diagnosis, tracking, and treatment. These biomarkers are obtained from many imaging modalities, including single-photon emission computed tomography (SPECT), positron emission tomography (PET), and magnetic resonance imaging (MRI). The effectiveness of a biomarker depends on the specific clinical question being investigated and on the stage of the disease. Neuroimaging biomarkers can help differentiate between several phases of PD, such as preclinical and prodromal, early, and moderate-to-late stages [53]. The major imaging targets and methods used, as well as their interventions subject to availability in clinical use, are discussed and summarized in Table 2.

#### 3.2.1. Imaging Strategies for PD Research and Diagnosis

##### Single-Photon Emission Computed Tomography (SPECT)

Single-photon emission computed tomography (SPECT) is a nuclear imaging technology that involves injecting a radioactive tracer into the patient’s body, such a tracer emitting single gamma ray photons. These gamma rays are detected by a rotational gamma camera and converted into light photons. The detected photons are utilized to create a three-dimensional picture of the radiotracer distribution in the body using mathematical algorithms. SPECT imaging gives useful information about many physiological processes and can be used to diagnose diseases, stage them, and evaluate therapy responses. SPECT tracers can be used for scintigraphy, which records a single projection picture of the tracer’s distribution inside the body [69].

##### Positron Emission Tomography (PET)

In Positron Emission Tomography (PET), a patient is given a radiotracer injection. The radiotracer releases positrons, which interact with electrons to produce two 511-KeV photons that are released in opposing directions. External detectors pick up these photons, which reveal details about the body’s radioactivity distribution. The annihilation photons are captured by rings of detectors in the PET scanners, enabling the localization of radioactive decay events. Based on the identification of these annihilation events, PET imaging helps with disease diagnosis and treatment by offering important insights into physiological processes [70].

##### Magnetic Resonance Imaging (MRI) and Functional Magnetic Resonance Imaging (fMRI)

Magnetic resonance imaging (MRI) relies on the interaction of hydrogen nuclei in the body with intense magnetic fields and radio waves. When a patient is placed in an MRI machine, their hydrogen atoms align with the magnetic field. Radiofrequency pulses are then used to cause the atoms to produce energy, which is measured by the MRI scanner. This radiated radiation is utilized to generate detailed photographs of the body’s internal structure using the distribution of the hydrogen atoms [71].

The idea behind functional magnetic resonance imaging (fMRI) is to estimate brain activity by measuring changes in blood oxygenation and flow. When neuronal activity increases in a brain area, neurovascular coupling causes localized changes in blood flow, volume, and oxygenation. fMRI detects the blood oxygenation level dependent (BOLD) signal, which is sensitive to variations in the proportion of oxygenated to deoxygenated hemoglobin in the blood. The magnetic characteristics of oxygenated and deoxygenated hemoglobin differ, allowing BOLD signal alterations to be directed at areas of increased cerebral activity, allowing for the mapping of brain function [72].

#### 3.2.2. Imaging Targets Used in PD

##### Dopaminergic System

Dopaminergic system imaging is an effective biomarker in the early stages of the medical condition and provides a means of evaluating neurodegeneration even in the preclinical and prodromal phases of PD.

##### Dopamine Synthesis

Certain neurotransmitters, which are substances that neurons utilize for communication, require the enzyme L-amino acid decarboxylase (AADC) in order to be produced. Dopamine synthesis specifically requires AADC, and a number of neurological diseases are marked by the aberrant function or loss of dopamine-producing neurons. Thus, monitoring AADC activity may provide information on how the disease is progressing [73]. FMT-PET (6-fluoro-meta-tyrosine positron emission tomography) is a high-resolution imaging technology used to directly measure the activity of the AADC enzyme in the brain. FMT-PET has been found to identify variations in AADC activity over time, a fact which may aid the prediction of PD in individuals suffering from rapid eye movement (REM) sleep behavior disorder (RBD). Longitudinal research has discovered that RBD patients who later developed PD or dementia with Lewy bodies exhibited substantially reduced AADC activity levels on follow-up FMT-PET scans compared to baseline [74]. F-DOPA PET detects the uptake of the F-DOPA tracer, which is decarboxylated to dopamine by the AADC enzyme. The reduced absorption of F-DOPA in PD patients shows altered AADC activity, which is a significant part of the disease process [75].

##### Dopamine Transporter

DAT imaging evaluates presynaptic dopamine deficits by targeting the DAT using positron emission tomography (PET) or single-photon emission computed tomography (SPECT) methods [76]. Both DAT-SPECT and DAT-PET imaging are useful methods for diagnosing dopamine insufficiency and for tracking the course of PD. While PET imaging offers a superior resolution and the potential for multimodal target evaluation, SPECT imaging is more widely used in clinical practice. In nonadvanced PD patients, the PET scan employing [18F] FE-PE2I has been shown to enable accurate assessment of the DAT deficit in several nigrostriatal areas, offering important insights into the course and severity of the disease. The putamen’s and sensorimotor striatum’s DAT availability has been found to be significantly correlated with scores on the Movement Disorder Society unified PD rating scale part III (MDS-UPDRS-III), especially when it comes to bradykinesia and rigidity symptoms. These findings validate the use of PET radioligand [18F]FE-PE2I as a marker of the severity of PD [77]. A recent study has shown that the diagnosis and clinical treatment of parkinsonism patients are significantly impacted by DAT-SPECT imaging. Following the findings of the DAT scan, 37% of patients experienced changes in diagnosis and 42% experienced changes in clinical treatment [78].

##### Vesicular Monoamine Transporter Type 2

VMAT2 is found in synaptic vesicle membranes and stores dopamine. VMAT2 is thought to be involved in monoamine absorption into synaptic vesicles. Two VMAT2 radioligands, [11C]DTBZ and [18F]FP-DTBZ, have been utilized to determine the degree of nigrostriatal deficit in PD patients using PET. Studies have revealed that VMAT2 radioligands such as [18F]FE-DTBZ-d4 have better stability and exhibit a better brain uptake capacity than other tracers, making them attractive for the assessment of VMAT2 in the brain [79].

##### Dopamine Receptors

Dopamine receptor imaging can reveal information about neurons on the postsynaptic side of nerve terminals, as most receptors are found on these membranes. Dopamine receptor imaging using SPECT and PET is critical for distinguishing multiple system atrophy (MSA) and progressive supranuclear palsy (PSP) from PD. These imaging methods can help assess the development rate of PD and aid the selection of suitable therapies, particularly with respect to individuals receiving neuroleptics [80].

##### Serotonergic System

The monoamine neurotransmitter serotonin is created from the amino acid tryptophan. It is then released into the synaptic cleft, stored in synaptic vesicles, and then re-captured by the corresponding transporter protein [81]. Compared to healthy controls, PD patients have fewer available 5-HT1B receptors in their brain. Creative ability and receptor availability are also associated, pointing toward a potential role of 5-HT1B receptors in the development of PD cognitive symptoms [82,83]. There is a correlation between the degree of apathy in prodromal PD and the integrity of the serotonin transporter signal in the dorsal raphe nucleus [84]. In both striatal and extra-striatal brain areas, the loss of serotonergic innervation is thought to be an early sign of PD [57].

##### Cholinergic System

LRRK2 mutation carriers, both healthy and suffering from PD, have greater cortical AChE activity compared to healthy controls and patients with idiopathic PD [85]. With PET and SPECT tracers, it is possible to scan this transporter. According to different studies, the above tracers show a considerably reduced availability of VAChT in the cortical areas of PD patients compared to healthy controls [86]. PD is characterized by substantial posterior cortical cholinergic degradation in nondemented individuals [87,88], with indications of progression from posterior to anterior over time [89]. GBA-mutated individuals with PD are more likely to experience early cognitive decline, as well as a higher reduction in cortical cholinergic denervation, even with normal cognition [90].

##### Noradrenergic System (NA)

The non-motor symptoms of PD may be caused by noradrenergic transmission deficits. In PD patients, noradrenergic activity decreases and is linked to cognitive impairment and RBD [91]. Prodromal PD does not show a significant decrease in NA transporters. However, there are some indications of substantially decreased availability in the sensorimotor cortex and thalamus [92]. Recent research suggests that NA receptors are reduced in both the motor insular cortex and the thalamus and putamen [93].

##### Cerebral Blood Flow and Glucose Metabolism

PD alters the normal functioning of the brain, including blood flow, oxygen and energy consumption, and interregional communication. The PET tracer [18F]-FDG can detect brain energy use [60].

Metabolic imaging with [18F]-FDG is able to identify PD-related metabolic patterns (PDRP) in PD patients. PDRP expression is assessed scan by scan, enabling an objective assessment of disease activity in individual patients, something which can help with the clinical evaluation and monitoring of PD development [61]. Recent research indicates that fMRI brain responses to DBS stimulation in PD patients might be an objective indicator of clinical responses [94].

##### Other Imaging Targets

A recent study has used structural brain scans to create and assess a robust explainable deep learning model for PD categorization in order to distinguish between PD patients and healthy controls. This study focuses on training a 3D convolutional neural network (CNN) utilizing one of the biggest collections of T1-weighted magnetic resonance imaging datasets [62]. Standard diffusion measures are not as accurate as free water (FW)-corrected diffusion measures. A recent study has assessed whole brain white matter and corrected diffusion metrics for these structures in patients suffering from PD, multiple system atrophy (MSA), and progressive supranuclear palsy (PSP). The study reports distinct differences in the patterns of FW alterations between PD and atypical parkinsonian disorders, indicating the potential use of whole brain FW maps as markers during the diagnosis of these disorders [95].

The transverse relaxation time (T2) of tissues is measured using T2 relaxometry in imaging to provide information on the composition and properties of the tissue. Iron sensitivity in imaging enables the identification and description of iron accumulation in tissues, especially in the substantia nigra (SN), using methods such as transverse MR relaxometry and quantitative susceptibility mapping (QSM) [96]. The advent of quantitative susceptibility mapping (QSM) has enabled the noninvasive quantification of brain iron levels in vivo via MRI scans, contributing to the understanding of iron-associated pathogenesis, and has the potential to contribute to the development of iron-based biomarkers of PD [97]. The distribution of neuromelanin and iron in the SN has been evaluated in a study using T2 relaxometry alongside iron-sensitive imaging. Such an approach allows for the visualization of the dorsal part of the substantia nigra pars compacta, which contains a considerable quantity of neuromelanin. Since increased iron deposition in PD patients can affect some imaging characteristics, such as the tail sign in nigrosome1, i.e., a cluster of dopaminergic neurons in the SN that contain neuromelanin, iron sensitivity affects how neuromelanin distribution is seen in imaging investigations [64]. Strong evidence suggests that neuroinflammation contributes significantly to the onset and development of PD and other neurodegenerative diseases. The activation of microglia plays a vital role in driving the inflammatory response of the brain [65]. PET ligands that target the 18 kDa translocator protein (TSPO) can be used to reveal neuroinflammation in vivo. When microglia are activated, there is a significant increase in the expression of TSPO, which is mostly expressed in the outer membrane of the mitochondria.

Numerous studies have documented a significant increase in microglial activation in PD patients [66]. There are recognized procedures for measuring the sympathetic denervation of the heart, such as SPECT or scintigraphy using iodine radio-labelled MIBG. The approach has been utilized to divide PD into two-to-three subcategories despite significant flaws [67,98]. The uptake of 18F-florzolotau has been found to be considerably greater in the cortical areas of patients suffering from PD-related dementia compared to PD patients with normal cognition and healthy controls, particularly in the temporal lobes. Notably, 18F-florzolotau uptake in the occipital lobe of individuals suffering from PD with dementia has been found to be significantly associated with cognitive impairment, as measured by mini-mental state examination (MMSE) scores [99]. According to logistic regression analysis, tau PET has a greater correlation with change management than amyloid PET across all participants and dementia types [100]. The α-synuclein PET tracer allows for the identification of prodromal and early-stage synucleinopathies and can provide information on the progression of the disease. It can also help differentiate parkinsonisms caused by synucleinopathies, tauopathies, and other diseases. However, no radiotracers have been approved for clinical use in humans [101]. The original magnetic resonance parkinsonism index (MRPI) is a useful technique for distinguishing between progressive supranuclear palsy-parkinsonism (PSP-P) and PD [102]. However, the MRPI 2.0 has shown improved diagnostic accuracy, with an area under the curve (AUC) of 0.91 with respect to the identification of these diseases in their early stages, something which is critical for a swift and accurate diagnosis. The MRPI 2.0 includes additional measures, such as the breadth of the third ventricle, which improve its sensitivity and specificity compared to the original MRPI. In recent investigations, it has demonstrated a sensitivity of 100% and a specificity of roughly 94.3% when distinguishing PSP-P from PD, making it a reliable tool for clinicians [103,104].

### 3.3. Genetic Biomarkers

Genetic biomarkers of PD are genetic variants related to the disease that can be found using genome-wide association studies (GWAS) and machine learning algorithms. These biomarkers can aid the diagnosis and progression monitoring of PD [105]. Recent advancements in genetic research methodologies have offered new perspectives on the role of the genetic background in the onset of PD. Therefore, PD is a multifaceted genetic condition triggered by a combination of aging, environmental factors, and inherited elements. There are two main approaches to identify genetic variables in PD patients. One approach consists of looking into unusual Mendelian variants of PD and finding the genes that cause them. The other entails the detection of risk variations via genetic statistical analyses of huge groups of subjects [106]. Research into Mendelian PD genetics has identified 24 genes or loci listed in the online Mendelian inheritance in man (OMIM) database that play a role in PD progression. The PRKN gene is responsible for autosomal recessive juvenile PD, while the leucine-rich repeat kinase 2 (LRRK2) variant causes autosomal dominant PD [106]. LRRK2 architecture impacts kinase activation, and increased LRRK2 substrate phosphorylation may contribute to PD etiology. Evidence suggests that oxidative stress and/or endolysosomal stress may increase LRRK2 kinase activity in idiopathic PD patients. Exposure to environmental toxins linked to idiopathic PD in epidemiological studies has also been shown to trigger LRRK2 kinase activity [107]. Through whole-exome sequencing, the vacuolar protein sorter-35 (VPS35) gene has been identified as the initial causal gene for autosomal dominant PD [108]. In a Japanese study, coiled-coil-helix domain containing 2 (CHCHD2) and prosaposin (PSAP) have been identified as novel genes associated with familial PD, albeit with relatively low prevalence in Mendelian PD [109,110]. DJ-1, FBXO7, SYNJ1, EIF4G1, GIGYF2, HTRA2, PLA2G6, DNAJC6, ATP13A2, and DNAJC13 are other families of PD-causing genes. However, the functions of these gene products, such as the management of oxidative stress, intracellular trafficking, phospholipid membranes, mitochondrial processes, and the ubiquitin–proteasome system, have all been associated with the development of PD. GBA1 variants associated with PD, such as p.E326K, p.T369M, p.N370S, and p.L444P, reduce the activity of glucocerebrosidase, the enzyme encoded by GBA1, thereby impairing the lysosomal breakdown of α-synuclein [111].

Despite the progress in genetic analysis technology and high-throughput methods, making new discoveries remains challenging. For instance, whole-exome sequencing of small PD families often reveals hundreds of candidate causative variants; however, typically, each family has only one causal variant, regardless of its size. Consequently, researchers must employ various strategies to reduce the number of potential variants from hundreds to a single one. It is estimated that at least 80% of the families with familial PD have a single candidate mutation, leaving the underlying gene unidentified (Table 3).

## 4. MicroRNAs and Peptides Show Potential to Be Next-Generation Biomarkers

MicroRNAs (miRNAs) and peptides are increasingly recognized as promising biomarkers of PD, with significant potential to improve early detection and diagnostic accuracy. A recent study has demonstrated the potential of circulating miRNAs as biomarkers for the detection of PD and associated disorders. A review by Giri et al. highlights that α-synuclein and miRNA can be detected in the urine of PD patients and can be used as early biomarkers in the diagnosis and prognosis of neurodegenerative diseases such as PD [125]. A meta-analysis has found that biofluid miRNAs may successfully differentiate between PD patients and healthy individuals [126,127]. Several studies have frequently associated specific miRNAs, including miR-24-3p, miR-146a-5p, and miR-331-5p, with PD, suggesting their potential as valuable diagnostic markers [127,128]. Previous research has demonstrated that the functional transfer of miRNAs via extracellular vesicles is being explored for its potential to detect not only PD, but also related disorders, such as random eye movement sleep behavior disorder, thereby enhancing its clinical relevance [129]. A study has found overexpression of miR-132 in plasma samples from male individuals suffering from PD compared to female patients, and it has also established the role of miR-132 in the regulation of the protein Nurr1, which affects dopamine regulation [130]. There are several other miRNAs, such as miR-7-1-5p, miR-223-3p (serum) [131], hsa-miR-144-3p (serum) [132], miR-24, miR-195, miR-19b (serum-EVs) [133], miR-22-3p, miR-10b-5p, and miR-151a-3p (CSF) [134], that are deregulated in different biofluids and that can be used as potential biomarkers for the early diagnosis of PD and for the monitoring of disease progression.

It has been reported that a panel of peptides can predict PD before the symptoms appear with an accuracy of 79%. This panel contains biomarkers such as the granulin precursor and the complement C3, both of which are associated with disease severity, indicating their potential in early detection and therapeutic use [135]. Another study has discovered cerebral fluid peptides that might successfully distinguish PD patients from healthy controls [136]. Innovative peptide-based methods are under development to precisely target risky α-synuclein oligomers. A plasmonic biosensor based on α-helical peptides has the potential to detect these oligomers early and improve diagnosis and therapy outcomes [137]. A variation of the small humanin-like peptide 2 (SHLP2) protein has been found to act as a protective factor against PD, lowering the risk by almost 50% in some groups. This finding highlights the significance of mitochondrial-derived microproteins in determining PD risk and paves the way for new treatment strategies [138].

## 5. Conclusions

Although Parkinson’s disease is caused by a combination of environmental variables and genetic predispositions, its diagnosis requires a multidisciplinary approach. Biomarkers are critical for improving our understanding of PD and its treatment. Identification and confirmation of biomarkers can lead to improved patient care, more accurate diagnoses, and better treatment outcomes.

In terms of early PD diagnosis, even before the clinical symptoms manifest, the α-synuclein seeding amplification assay (αSyn-SAA) represents a significant advancement. GFAP may be a valuable biomarker for predicting cognitive decline and dementia conversion in PD patients. In prospective cohort studies, combining blood biomarkers for the prognosis and diagnosis of PD can increase diagnostic accuracy. The diagnosis of PD can be rendered more sensitive and specific by choosing the right signs for combination detection. The dopaminergic system is now the most widely accessible, well-established, and validated biomarker in clinical practice. Understanding the genetic variables affecting PD risk, onset, and development is critical to develop therapies that can reduce disease progression. Numerous genes and GWAS sites have been linked to the development of PD. We need to continue the investigation into possible genetic risk factors and work together to grasp their implications at the molecular and biological level. Understanding genetic and functional information is crucial for developing proper treatments for PD. Correlating imaging data with biochemical markers can increase PD diagnosis accuracy. It can also help assess therapy success, particularly during the early stages when neuroprotective medications are most beneficial. This literature review highlights the continuous efforts to identify new indicators, improve the efficacy of existing biomarkers, and close the diagnostic gaps. With further research, the application of accurate biomarkers in clinical practice has the potential to transform the way PD is managed, improve the accuracy of diagnosis, and, ultimately, benefit patients. In addition to enriching our present knowledge of PD, this review highlights interesting directions for further research and advancement in the hunt for efficient preventative and treatment methods.

## 6. Future Prospects

Future research should combine biochemical, neuroimaging, and genetic markers to provide a complete biomarker profile of PD. This multimodal method may increase diagnostic accuracy and assist early diagnosis by capturing the disease’s multidimensional character. Longitudinal studies that investigate biomarker changes over time are needed among individuals at risk of developing PD or in the early stages of the disease. Investigating gene–environment interactions might provide crucial insights into PD etiology. Despite these advancements, the establishment of a comprehensive biomarker panel specifically for PD remains a focus for future research [127]. Large-scale proteomics techniques have revealed possible diagnostic markers in serum samples, including ALCAM, contactin 1, CD36, DUS3, NEGR1, Notch1, TrkB, and BTK, all of which correlate with clinical scores and disease progression [139]. Additionally, employing a neurology 4-plex-A biomarker panel alongside plasma α-synuclein has demonstrated potential with respect to the differentiation of PD patients from healthy individuals and with respect to the prediction of clinical disease progression [140]. When combined with clinical factors, blood biomarkers such as serum neurofilament light (NfL) and genetic status (GBA and APOE) have been shown to be beneficial in PD prognostic modeling, offering a stronger prediction of poor outcomes [141]. Furthermore, extracellular vesicles (EVs) produced from PD patients have shown promise as a source of novel biomarkers for diagnosis, prognosis, and therapeutic monitoring [142].

## Figures and Tables

**Table 1:** Lists of the different biochemical biomarkers used in the diagnosis of Parkinson’s disease, describing recent findings on their role with respect to PD, their prospective use, and their limitations at the onset of PD diagnosis. These biomarkers aid the understanding and diagnosis of Parkinson’s disease owing to their distinct roles in the neurophysiological processes and pathological changes linked to the condition.

Biochemical Biomarkers	Findings Relative to PD	Prospective Use	Limitations	References
α-synuclein	The α-synuclein seeding amplification assay (αSyn-SAA), is a very accurate method for identifying aberrant α-synuclein in spinal fluid.	Early PD detection with the αSyn-SAA test has demonstrated encouraging results. In [32], it was able to determine that the disease was absent from 96% of persons without PD and that it was present in 87% of the participants with PD.	Variations within the result. Additional validation is needed.	[32]
Orexin	Orexin-A promotes dopaminergic neuron survival and function by acting on orexin receptors. Changes in orexin levels may have a role in the pathophysiology of nonmotor symptoms.	Neuroprotective role. Effect on sleep disturbances.	Intricate interactions with other neurotransmitters.	[20,21]
Uric acid	Low serum UA levels may correlate with the severity of sleep disorders in PD patients and act as a biomarker of poor sleep quality in PD patients.	Risk factor identification. Disease progression monitoring. Nonmotor symptom assessment. Therapeutic targets.	Lack of mechanistic understanding.	[43,44,47]
Tau protein	In vitro, tau-modified α-synuclein fibrils show higher seeding activity than pure α-synuclein fibrils, resulting in mitochondrial malfunction, synaptic impairment, and neurotoxicity. Tau-PET imaging enables the visualization and measurement of tau load in the brain, offering insights into the distribution and impact of tau pathology across PD patients.	Influence on its pathophysiology and contribution to disease progression. Potential therapeutic target.	Variability, specificity, and sensitivity.	[45,46,48]
Protein DJ1	Mutations in PARK7 result in nonfunctional DJ-1 protein or in their total loss. In general, PARK7/DJ-1 dysfunction significantly impact the immunological responses and neuroinflammatory processes of microglia.	Prospective therapeutic target.	Complexity of pathogenesis. Limited understanding of the mechanisms.	[41,42]
GFAP	Prognostic biomarker of dementia conversion in patients with PD who have mild cognitive impairment.	Tracking the development of the disease. Conversion of motor subtypes in early PD.	Lack of specificity.	[49,50,51]
Apo-A1	Apo-A1 is a prominent blood biomarker associated with PD and moderate cognitive impairment (PD-MCI). Lower levels of Apo-A1 in PD patients raise issues regarding its potential neuroprotective properties and participation in the etiology of PD.	Diagnostic marker. Therapeutic target. Risk assessment and prognostic value.	Single biomarker complexity. Specificity, sensitivity, and variability. Clinical translation challenges. Complexity of lipid metabolism.	[36,37]
8-OHdG	Specific gene mutations have been linked to higher levels of 8-OHdG in PD patients. 8-OHdG is related to the degradation of dopaminergic neurons in PD.	Valuable biomarker for assessing oxidative DNA damage. 8-OHdG measurements can be used to explore the impact of genetic variants on oxidative DNA damage and susceptibility to PD.	Interpretation of 8-OHdG levels can be complicated. The fluctuating nature of 8-OHdG levels could complicate the assessment of disease progression. Lack of specificity.	[29]
Aβ42	The APOE genotype may alter amyloid-β-42 levels in CSF, indicating a more vulnerable subtype of PD characterized by significant amyloidopathy in the early stages. Plasma extracellular vesicle (EV) tau, β-amyloid, and α-synuclein levels in PD patients are associated with clinical progression, motor and cognitive deterioration, and postural instability in affected individuals.	Prediction of clinical outcomes. Monitoring of disease progression. Early-stage detection of disease.	Lack of specificity.	[40,52]

**Table 2:** Potential applications of neuroimaging biomarkers in the context of PD.

Imaging Target	Modality	Interventions in the Context of PD	Available for Clinical Use	References
Dopamine synthesis Dopamine transporter	PET, SPECT	Quantifiable loss of dopaminergic terminals. Detecting striatal neuron loss in MSA and PSP, quantifying dopamine release, and determining receptor occupancy using D2/3-targeting anti-PD drugs.	Established and broadly available.	[54,55,56]
Serotonergic system	PET	Determining whether the raphe nuclei have lost any serotonergic nerve terminals.	Not established and not available.	[57,58]
Cholinergic system	PET, SPECT	VAChT loss observed in the cortex. AChE depletion has been observed in both cortical and peripheral nerve systems. A decrease in αβ-42 has been seen throughout the brain.	Not established and not available.	[57]
Noradrenergic system	PET, SPECT	Norepinephrine transporter loss detected in the midbrain and thalamus. Identifying loss of cardiac noradrenergic nerves.	Not established and not available.	[59]
Glucose metabolism	FDG-PET	Identifying metabolic, blood flow, and functional connectivity characteristics associated with PD.	Not established and not available.	[60,61]
Brain volume and atrophy	T1-weighted structural MRI	This structural MRI method provides comprehensive brain structural information and is helpful in distinguishing PD from secondary and atypical types of parkinsonism.	Established and broadly available.	[62]
Iron in the brain	Iron-sensitive MRI (T2* relaxometry)	The amount of iron accumulation in the substantia nigra of PD patients is correlated with the severity of their motor symptoms, and T2* relaxometry can identify elevated iron levels in this region when compared to controls.	Established and broadly available.	[62]
Free water imaging	Diffusion MRI	The understanding of structural alterations in PD patients can be aided by MRI methods that evaluate tissue integrity and microstructures, especially in areas like the substantia nigra.	Not established and not available.	[63]
Neuromelanin	MRI	This MRI method provides information about changes in the substantia nigra, a region affected by PD, by visualizing the neuromelanin content in this area.	Not established and not available.	[64]
Cerebral blood flow	PET, SPECT, fMRI	It is helpful to distinguish PD from secondary and atypical types of parkinsonism.	Not established and not available.	[65]
18Kd translocator protein	TPSO-PET	It provides information about microglial activation and neuroinflammation in PD patients.	Not established and not available.	[66]
123I-metaiodobenzylguanidine (123I-MIBG)	SPECT/scintigraphy	It has a correlation with the clinical profile and severity of PD. It can identify cardiac sympathetic denervation with greater sensitivity.	Established and available.	[67]
Beta amyloid	PET	Patients with PD dementia (PDD) frequently have significant deposits of amyloid-β (Aβ) which contribute to memory loss and accelerate the rate of cognitive impairment.	Established and available.	[68]
Tau	PET	Tau-PET imaging studies have identified aberrant tau deposition in the brain of PD patients with cognitive impairment (PDCI), including PD dementia (PDD) and PD mild cognitive impairment (PD-MCI).	Established and available.	[46]

T2*: observed or effective relaxation time.

**Table 3:** Genetic biomarkers and their potential roles in the context of PD.

Biomarker	Chromosome Location	Characteristics	Mutations	Pathological Features	References
LRRK2	12q12	Contains GTPase and autosomal dominant.	R1441G, R1441C, R1441H, Y1699C, G2019S, R1628P, G2385R and I2020T	It can cause dopaminergic neuronal cell death, reduced dopamine neurotransmission, and protein synthesis and degradation problems.	[112]
PINK1	1p36.12	Autosomal recessive, with eight exons encoding a 581 amino acid serine/threonine protein kinase.	p.Gln456Stop, p.Leu347Pro	Hypereflexia, dystonia, early l-dopa-induced dyskineasis, and mental and behavioral difficulties can occur.	[113,114]
PRKN	6q25.2-27	G403C mutations cause a glycine to arginine amino acid change.	G403C, deletion mutation of exon 6	Exon 6 deletion mutations cause EOPD.	[115,116]
UCH-L1	4p13	Autosomal dominant.	p.Arg178Gln, p.Ala216Asp	Visual impairment, impaired light perception, optic atrophy, and more.	[117,118,119]
SNCA	4q21.3-q22	SNCA is a sarcopterygian-specific gene. Five exons encode this autosomal dominant gene.	A30P, E46K, H50Q, G51D, A53T	Lewy body dementia is characterized by cognitive fluctuations, visual hallucinations, REM sleep behavior, and other symptoms.	[120,121,122]
VPS35	16q12	Autosomal dominant.	D620N	The mutation impairs LAMP2a retrieval from the endosome to the Golgi, resulting in a drop in LAMP2a levels within neuronal cells. This induces an increase in α-synuclein buildup within these cells.	[123]
GBA	1q21	Autosomal recessive.	N370S and L444P	GBA mutations cause mitochondrial and lysosomal dysfunction.	[124]

## Data Availability

No new data were created or analyzed in this study.
